# The use of drones for mosquito surveillance and control

**DOI:** 10.1186/s13071-022-05580-5

**Published:** 2022-12-16

**Authors:** Gabriel Carrasco-Escobar, Marta Moreno, Kimberly Fornace, Manuela Herrera-Varela, Edgar Manrique, Jan E. Conn

**Affiliations:** 1 Health Innovation Laboratory, Institute of Tropical Medicine “Alexander Von Humboldt”, Universidad Peruana Cayetano Heredia, Lima, Peru; 2 School of Public Health, University of California San Diego, La Jolla, USA; 3 Faculty of Infectious and Tropical Diseases and Centre for Climate Change and Planetary Health, London School Hygiene and Tropical Medicine, London, UK; 4 School of Biodiversity, One Health and Veterinary Medicine, University of Glasgow, Glasgow, UK; 5 Grupo de Investigación en Entomología, Facultad de Medicina, Universidad Nacional de Colombia, Bogotá, Colombia; 6 The Wadsworth Center, New York State Department of Health, Albany, NY USA; 7 Department of Biomedical Sciences, School of Public Health, State University of New York, Albany, NY USA; 8 Saw Swee Hock School of Public Health, National University of Singapore and National University Health System, Singapore, Singapore

**Keywords:** Drones, Uncrewed aerial vehicle, Malaria, Dengue, Control, Infectious diseases

## Abstract

In recent years, global health security has been threatened by the geographical expansion of vector-borne infectious diseases such as malaria, dengue, yellow fever, Zika and chikungunya. For a range of these vector-borne diseases, an increase in residual (exophagic) transmission together with ecological heterogeneity in everything from weather to local human migration and housing to mosquito species’ behaviours presents many challenges to effective mosquito control. The novel use of drones (or uncrewed aerial vehicles) may play a major role in the success of mosquito surveillance and control programmes in the coming decades since the global landscape of mosquito-borne diseases and disease dynamics fluctuates frequently and there could be serious public health consequences if the issues of insecticide resistance and outdoor transmission are not adequately addressed. For controlling both aquatic and adult stages, for several years now remote sensing data have been used together with predictive modelling for risk, incidence and detection of transmission hot spots and landscape profiles in relation to mosquito-borne pathogens. The field of drone-based remote sensing is under continuous change due to new technology development, operation regulations and innovative applications. In this review we outline the opportunities and challenges for integrating drones into vector surveillance (i.e. identification of breeding sites or mapping micro-environmental composition) and control strategies (i.e. applying larval source management activities or deploying genetically modified agents) across the mosquito life-cycle. We present a five-step systematic environmental mapping strategy that we recommend be undertaken in locations where a drone is expected to be used, outline the key considerations for incorporating drone or other Earth Observation data into vector surveillance and provide two case studies of the advantages of using drones equipped with multispectral cameras. In conclusion, recent developments mean that drones can be effective for accurately conducting surveillance, assessing habitat suitability for larval and/or adult mosquitoes and implementing interventions. In addition, we briefly discuss the need to consider permissions, costs, safety/privacy perceptions and community acceptance for deploying drone activities.

**Graphical Abstract:**

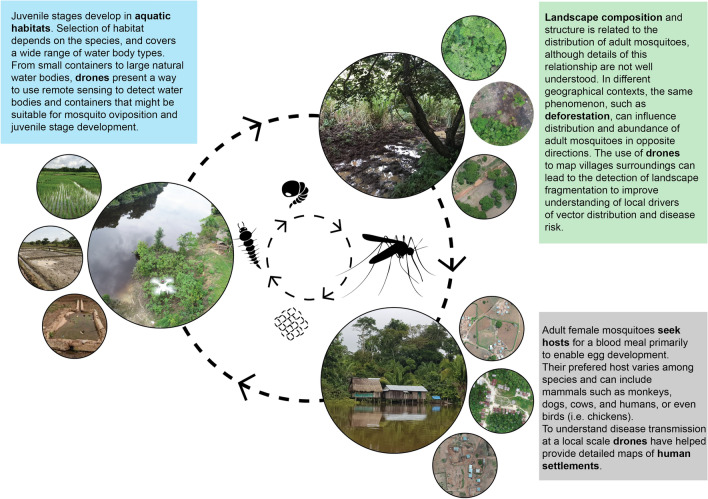

**Supplementary Information:**

The online version contains supplementary material available at 10.1186/s13071-022-05580-5.

## Background

### New tools for mosquito control and rationale

A main driver of the need for new, more effective mosquito surveillance and control methods is increased insecticide resistance across multiple vector species of *Aedes*, *Anopheles* and *Culex* [1]. Resistance has led to the re-examination of interventions specific to different mosquito life stages, such as larval source management, that might be considered to be a component of integrated vector control. Other drivers include the need to tailor interventions for diverse local landscapes and to engage human communities more directly. For malaria, an increase in residual (exophagic) transmission together with ecological heterogeneity in everything from weather to local human migration and housing to mosquito species’ behaviours presents many challenges [2]. Ironically, higher insecticide resistance [1] is partly a result of the intensive roll-out of long-lasting insecticidal nets (LLINs), sometimes combined with indoor residual spraying (IRS), which reduced malaria cases successfully from 2000 to 2015 in many malaria-endemic countries [3, 4].

In recent years, global health security has been threatened by the geographical expansion of arboviruses such as dengue and yellow fever and emergent viruses such as Zika and chikungunya [5]. The increased range of competent *Aedes* mosquitoes worldwide has been linked to global travel, urbanization modifications and environmental changes [3, 6, 7]. Resistance to insecticides employed both for larvae and adult control is prevalent in *Aedes* [8, 9] and *Culex*, compromising effective control of dengue and other arboviruses [10] and of lymphatic filariasis [11, 12]. The global landscape of mosquito-borne diseases and disease dynamics fluctuates frequently, and there are likely to be serious public health consequences for the success of mosquito surveillance and control programmes if issues of insecticide resistance and outdoor transmission are not adequately addressed.

A major complication of residual transmission in malaria endemic regions is locating the species and populations of questing and resting vectors that are outside houses [13]. Those that feed and/or rest in the peridomestic area, including in and around animal enclosures, may be vulnerable to interventions such as insecticide-treated barrier screens (with the obvious caveat of local insecticide resistance), spatial repellents or endectocide treatment of domestic animals [14]. Depending on local/regional ecology and human behaviour, it may be effective to detect and target aquatic stages. For controlling both aquatic and adult stages, remote sensing data have been used together with predictive modelling for risk, incidence and the detection of transmission hot spots and landscape profiles in relation to mosquito-borne pathogens for several years [15, 16]. Treatment of transmission hot spots can be effective in reducing—possibly in eliminating—arthropod-borne pathogens, but in low transmission settings, targeting the household level (the smallest unit), or even the community, may not translate to nearby non-targeted areas [17].

**Fig. 1:**
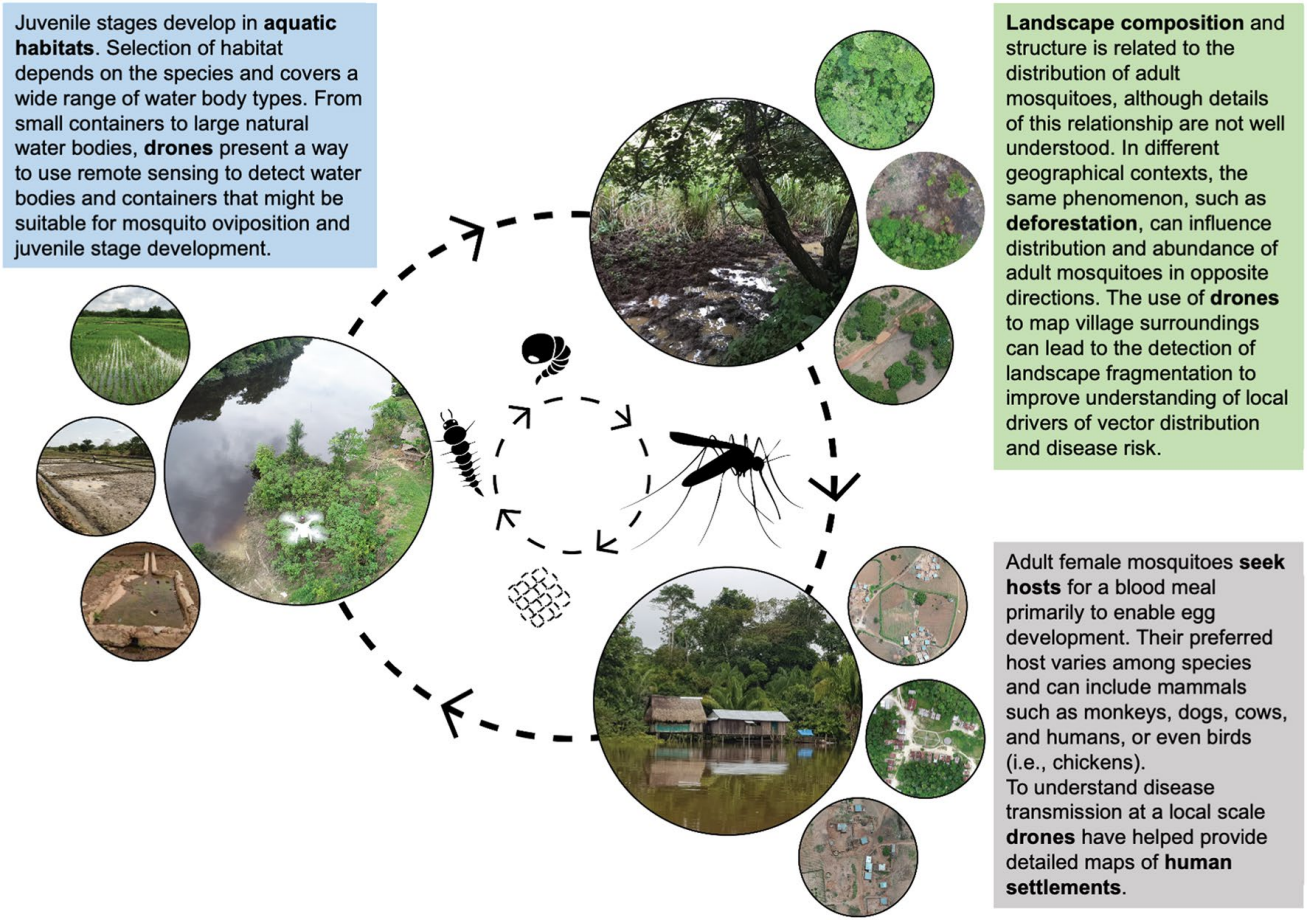
To understand the different stages at which drones can be used in surveillance and control of vector-borne pathogens it is important to follow the vector life-cycle and relate it to the environment wherein mosquitoes develop. Juvenile stages develop in aquatic habitats; in contrast, adult mosquitoes are broadly dispersed in the landscape. Drones are being used to produce high-resolution maps of these landscapes and to assist surveillance and control activities in the field

Here we outline the opportunities and challenges for integrating drones into vector surveillance and intervention strategies across the mosquito life-cycle (Fig. 1). We present a five-step systematic environmental mapping strategy that we recommend be undertaken in locations where a drone is expected to be used, outline the key considerations for incorporating drone or other Earth Observation data into vector surveillance and provide two case studies of the advantages of using drones equipped with multispectral cameras: (i) to locate anopheline vector breeding sites in Amazonian Peru; and (ii) to map fine-scale deforestation or other environmental modification that affect wildlife movements. We describe in some detail a series of pre-flight considerations that include local and regional drone regulations, privacy, safety, community engagement and feasibility. To illustrate the range of practical applications, we discuss examples of mapping breeding containers of juvenile aedine mosquitoes, and preliminary ongoing intervention studies to enable the use of drones to disperse biological control agents, chemical larvicides, *Wolbachia*-carrying mosquitoes or sterile males. Included in the Additional file 1: Text 1 is a glossary. We encourage serious consideration of high-resolution drone imagery data using specific sensors that can be incorporated into predictive distribution models and environmental suitability analysis to assess the risk of establishment and current and future spread of mosquito-borne pathogens.

### Water bodies and breeding site detection

Aquatic stages of mosquitoes are amenable to the use of drones (also known as umanned aerial vehicles [UAVs]) for identification, surveillance and treatment. Generally, container breeders, such as *Aedes aegypti* and *Aedes albopictus*, oviposit in many kinds of water-filled receptacles [18, 19], whereas non-container breeders, such as *Culex* species, are associated with stagnant water rich in organic matter [12], and *Anopheles* species choose natural (ponds, puddles, river edges, swamps) or anthropogenic water bodies such as fish-ponds, irrigated rice fields and mining ponds. Preferred aquatic site profiles have been established for many individual vector species (e.g. *Anopheles dirus*, *Culex quinquefasciatus*, *Ae. albopictus*) that are essential for both ground truthing and training remote sensing tools to enable detection of specific types of breeding sites.

**Fig. 2:**
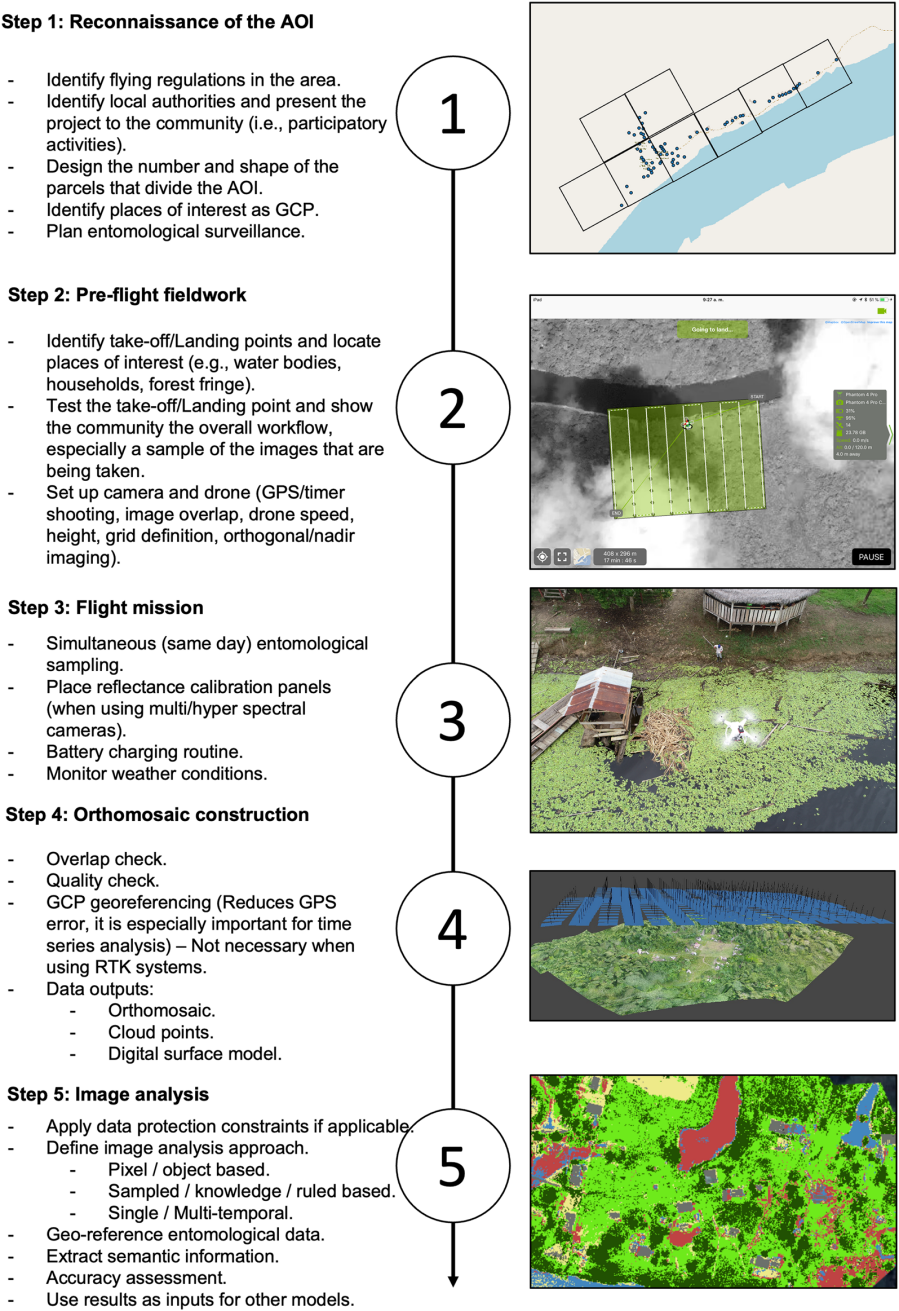
To successfully map the environment where mosquitoes develop it is important to do it in a systematic way, to guarantee repetition on subsequent sampling campaigns and to achieve the proposed goals of the survey. Steps 1–5 show an example of the workflow that is used to map the aquatic habitats of anopheline mosquitoes. AOI, Area of interest; GCP, Ground Control Points; GPS, Global Positioning System

Commercial drones have been used for surveying potential breeding sites in contrasting environments (rice fields, ponds and urban and peri-urban areas in Zanzibar, Tanzania), as demonstrated for malaria vectors by Hardy et al. [20]. In a tidal marsh in California (USA), Haas-Stapleton et al. [21] used a drone fitted with a multispectral camera to map accumulated surface water with the objective of prioritizing sites for larval stage mosquito inspection and to improve drainage ditches to reduce mosquito oviposition. This study also tested the capacity of a drone with a high-magnification camera to visualize immature mosquito stages of *Culex pipiens* from varying heights and colour-contrasted containers, ultimately demonstrating its potential to estimate mosquito abundance that would reduce work effort in mosquito habitat ground surveys. In Amazonian Peru, water bodies positive for the presence of mosquito larvae of the malaria vector *Nyssorhynchus darlingi* were identified with an overall classification accuracy > 85% by drones fitted with multispectral cameras ([22]; see section Case study 1). In a challenging intertidal coastal saltmarsh landscape in South Australia, researchers were able to map areas of shallow inundation among hummocks of low vegetation, establishing a useful reflectance threshold with an overall classification accuracy of 80% to detect putative breeding sites with a minimum depth of 10 cm, of local *Aedes* vectors of the Ross River virus [23]. A brief description of the workflow for drone survey is presented in Fig. 2.

### Spatial distribution of adult stages and abundance

Mosquitoes, like many organisms, are aggregated by available resources (e.g. resting and oviposition sites, hosts, sugar sources) within the landscape of their geographical range [24, 25]. Reisen [26] recognized specific habitat characteristics of importance in predicting mosquito abundance and species composition. Vegetation indices, land use, canopy cover, elevation and hydrology can have significant effects on the availability of aquatic habitats suitable for immature stages or resting areas for adult mosquitoes. Heterogeneous micro-environmental factors associated with host availability around houses can also have a significant influence on the spatial distribution of adult malaria vectors [27]. For example, the number of people sleeping in the dwelling, an individual’s relative attractiveness to mosquitoes, house construction materials, availability of aquatic habitats and distance to aquatic environments can all shape anopheline distribution. In other mosquito species, such as *Culex territans* and *Culex peccator*, the distribution of host animals within the landscape is of paramount importance to explain mosquito spatial distribution [28].

Characterization of land cover/use with remote sensing data proved to be practical to estimate the diversity and abundance of adult *Anopheles* for the study of mosquito habitats at a local scale and subsequently to design appropriate sampling strategies that represent the heterogeneous environment in French Guiana [29]. More sophisticated analysis using light detection and ranging (LiDAR) technology has been able to detect and classify fine-scale and temporal distribution of malaria vectors by the difference in their wingbeat frequencies compared with those of other insects, and between male and female mosquitoes [30].

## Pre-flight considerations

Despite the utility of drones for vector surveillance and control, programmes need to consider several relevant aspects, including local or regional regulations, safety, privacy and ethics, community acceptance and, as a final reality check, feasibility.

### Drone regulations

Prior to flying a drone, the first step is to review and understand local regulations for the surveillance location proposed. Authorizations for equipment, pilot and the activities to be conducted with the drone, as well as type and characteristics of the drone (i.e. weight or number of rotors) vary between countries [31]. Generally, countries have been slow to establish national drone regulations, thereby hindering widespread utilization of this technology [31]. Although a comprehensive analysis of drone regulations has been published [31], both the technology and its regulation continue to change rapidly.

### Safety

As the popularity and volume of operations with drones increase, concerns have been raised about drone safety and security. In contrast with other aerial vehicles, the only subjects at potential risk of a crash accident are people on the ground. Hence, new frameworks and strategies have been developed and are continuously being updated. Overall, the field is changing from crash-free to safe-to-crash operations addressed by software- and hardware-based solutions [32]. This is of particular importance for drones operating above populated areas. As will be discussed further in this review, drones could be used to monitor water bodies as potential sites for mosquito oviposition (an example of an un-populated scenario), as well as risk mapping of dwellings located along an environmental gradient (populated scenario). In this context, detect-and-avoid systems have received particular attention [33]. Pham et al. [34] and Shakhatreh et al. [33] reviewed major categories of collision avoidance methods that can be summarized as geometric, path planning, potential field approaches and vision-based approaches. Future scenarios that involve the simultaneous use of multiple drones require additional detect-and-avoid strategies called swarm intelligence algorithms [35] that are able to combine data from diverse sources, including location and weather sensors, accelerometers, gyroscopes, radio detection and ranging systems (RADARs) and LiDAR technology [33].

### Privacy and ethical issues

Valid privacy concerns have emerged from the use of drones, particularly by the unprecedented spatial and temporal resolution at which data collection can be conducted. Recent sensors mounted on drones can photograph at sub-centimeter resolution in comparison to the 5- to 10-cm resolution from private or military satellites, or 5- to 10-m resolution from freely available satellite images [36]. In many countries there are legal requirements for data collection from private property using drones; however, in rural areas or marginalized populations (where most tropical diseases are transmitted), clear distinctions of private property are often lacking [37]. Drones can (intentionally or unintentionally) collect private information directly, such as data on location, behaviour, body characteristics and/or use of space, or indirectly, such as on income, habits or family composition, raising ethical concerns about restriction of freedom and privacy violation [37, 38]. Despite the increasing use of drones in biomedical and, particularly, in infectious diseases research, institutional guidelines and relevant permits are still scarce or in their infancy. Ethical and best practices described by Hodgson and Koh [39] could be adapted for these scenarios. These practices include: (i) adopt the precautionary principle in lieu of evidence; (ii) utilize the institutional ethics process to provide oversight to drone research; (iii) adhere to relevant civil aviation rules and adopt equipment maintenance and operator training schedules; (iv) select appropriate drone and sensor equipment to minimize disturbance to the population of interest; and (v) exercise minimum disturbance flight practices and stop operations if they are excessively disruptive.

### Community acceptance

In rural settings, in addition to authorization to fly drones, an important aspect to consider is the community acceptance of such activities. A difference with other remote sensing instruments (such as satellites) is that drones fly in the aeronautic space (thus national aeronautic authorization is required) and are perceivable by the population in the study area [40]. Even though the area of interest (AOI) of the flight plan cannot include dwellings, drone operations may be perceived as a violation of privacy, a concern that must be recognized by the research team [41]. Prior to data collection, community meetings should be held to engage the local population and inform people about study objectives, benefits and the best practices that will be conducted (i.e. blurring personal information, such as license plates and faces; displacement; jittering; micro-spatial aggregation) to preserve privacy [36, 42]. In urban areas, other aspects may include noise, design or patterns of movement of the device [43, 44].

### Feasibility and equipment

Considering the large variety of drone models [45] and specifications, it is important to consider the feasibility of the intended applications (i.e. mapping, surveillance, control). The two main drone types are multi-rotor and fixed-wing. Multi-rotor drones are suitable for capturing stable images because of their precision control over position and tilt; however, the energy consumption of their rotors impacts the flight time and speed and limits their use for long-distance inspection and monitoring over extended periods. On the other hand, the aerodynamic design (similar to airplanes) of fixed-wing drones provides efficient use of energy for longer flight times and greater speed. The main limitation of fixed-wing drones is the requirement of large, clear areas for take-off and landing (in contrast to multi-rotor drones, which are able to take off and land vertically). In settings with dense forest cover, such as in the Amazon or Southeast Asia [22, 46], multi-rotor drones might be suitable, and in areas of, for example, open savanna, fixed-wing drones might be the most appropriate. Alongside the selection of drones is the cost of the equipment and sensors. Commercial multi-rotor drones range in cost from US$1000 to US$10,000, and fixed-wing drones from US$5000 to US$20,000, all commonly equipped with RGB (red, green and blue) cameras. It is important to consider the additional cost of supplementary sensors, such as multispectral, thermal or LiDAR sensors, which range in cost from US$5000 to US$20,000. Additional costs include spare parts for field surveys, such as controllers, extra batteries, propellers, landing paths, data storage, charging supplements, repair kits, mounting kits and packaging, as well as costs for personnel, field survey costs and analysis and computing requirements. While costs for drones and sensors are becoming increasingly affordable, drone survey plans should include a budget that covers other associated costs.

## Integrating drones into vector surveillance

Surveillance is defined as “the continuous and systematic collection, analysis and interpretation of disease-specific data and the use of those data in the planning, implementation and evaluation of public health practice” and has been recognized by the WHO as a core intervention for vector-borne disease control [47].

### Surveillance

A key surveillance priority is monitoring the distribution and abundance of local vector species to evaluate potential impacts on disease transmission. This requires keeping track of vector habitats and assessing changes which may increase or decrease vectorial capacity directly (e.g. landscape change that creates a suitable habitat) or indirectly (e.g. increasing availability of blood meals, resting sites or predation) or leads to ecological shifts in biting behaviour or the predominant vector species [48]. Entomological surveillance is essential for monitoring and evaluating interventions, identifying priority areas for further surveillance and assessing vector-borne transmission potential, and for determining whether migration of infected people or vectors into an area or the replacement of one vector species by another is likely to lead to disease outbreaks. Generating accurate maps of mosquito breeding sites might also provide an opportunity to use them as information, education and communication materials to display specific risk conditions for a given locality [47].

### Earth observation

**Fig. 3:**
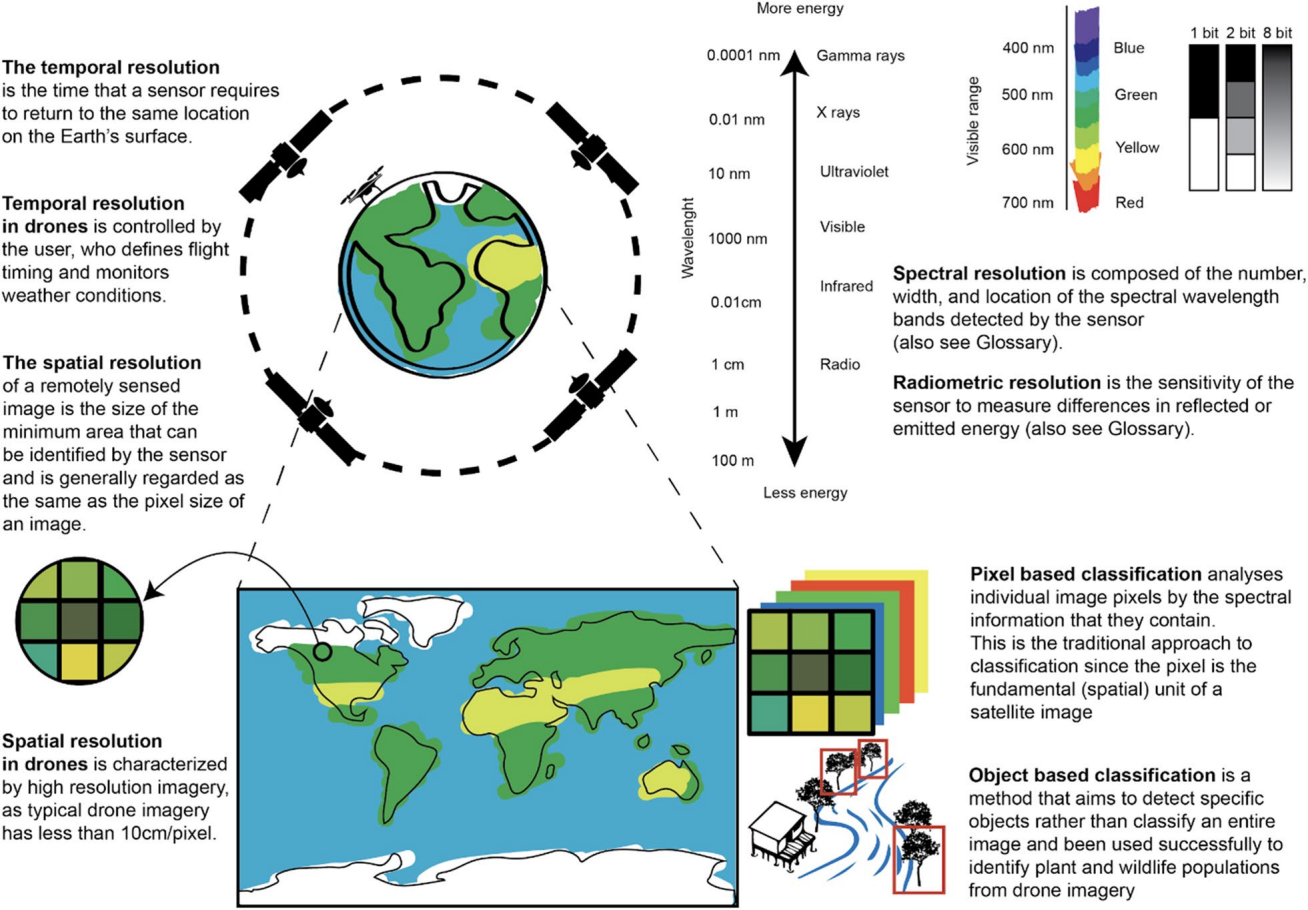
Frequently used remote sensing definitions

Earth observation (EO) is the collection of information on the physical characteristics of Earth’s surfaces using remote sensing technology, such as satellites, manned aircraft or drones. Raster data from EO are used frequently to characterize vector habitats and to monitor changing risks [49]. EO uses sensors that measure reflectance or emitted radiation from a distance, with wavelength interval size and number of wavelengths measured on the electromagnetic spectrum defining the spectral resolution of the sensor [50]. These sensors are also characterized by radiometric resolution, i.e. the sensitivity of the sensor to measure differences in reflected or emitted energy. While RGB sensors measure only the visible range of the electromagnetic spectrum (similar to standard cameras), sensors with higher spectral resolution increase the capacity to detect specific habitat types and allow transformations, such as for vegetation indices. Additionally, EO data are characterized by spatial resolution, which is the size of a pixel within the image, and by temporal resolution, which is the frequency at which the data are collected. While satellite data often have much higher spectral resolution than data collected by drones, spatial resolution of satellite data is typically more constrained because freely available data are limited to a resolution of ≥ 20 m/pixel; higher resolution data are often prohibitively expensive. In contrast, typical aerial imagery collected by drones has a resolution of < 10 cm/pixel (Fig. 3). Additionally, drones offer distinct advantages for temporal resolution as the user is able to define flight timing, whereas satellite data are only available when a satellite is passing over a specific location and may be further limited by cloud cover obscuring the target site [46]. Despite these advantages, drones cover much smaller geographical areas than satellite imagery and satellite data are more appropriate when large regions are targeted. For example, typical applications of low-cost drones result in < 1 km^2^ of area being mapped per 30-min flight due to different reasons, such as battery life and legal permission from Aviation Authorities (weight, visual line of sight [VLO], speed and maximum altitude) [46]. While the area covered will depend on local conditions, types of drones used and the flight height, commercially available drones are only able to cover limited geographical areas. Due to this limitation, drones are frequently best used to complement existing sources of EO data (Fig. 3). This may include drone use to conduct targeted mapping of high priority areas identified by satellite-based EO data or, conversely, using drones to collect training data to classify satellite EO data.

Data sources and analysis methods to be used should be determined by the features to be identified and the resolution required for surveillance; for example, direct observation of larval breeding sites at set time intervals for targeted larval source management. In other cases, the objective may be monitoring landscape changes or estimating numbers of people at risk or potential disease hosts. Based on these features and the technical capacity available, a specific approach to analyse drone data should be determined. This approach can include the following steps:

- Manual digitization: the simplest approach that relies on visual identification of key features; this has been shown to be effective for pinpointing malaria vector breeding sites in Tanzania [20].
- Region-growing/technology-assisted digitizing: this approach builds on manual digitization by using automated approaches to classify similar neighbouring pixels [51].
- Unsupervised classification: these methods group features within an image into different categories without the use of training data to define classes of interest. Such methods are used less frequently alone as they require the user to determine subsequently what the categories represent.
- Supervised classification: methods that use training data to fit a model classifying the imagery into pre-defined categories of interest. Training data may include specific categories or use ground-based data for imagery analysis. An example of pixel-based supervised classification is found in section Mapping juvenile aquatic habitats (Case study 1).
- Object detection: these methods aim to detect specific objects, such as plants and wildlife, rather than to classify an entire image [52, 53].

While a wide range of different models can be used for all approaches, machine learning methods are being utilized increasingly for image analysis. These can be implemented in a range of open source and commercially available software, with additional cloud-based computing power available through online platforms such as Google Earth Engine and Amazon Earth. Additional considerations should include the technical skills of the data analyst; while manual digitization and region-growing approaches can be used by non-experts, significant technical expertise is required to implement machine learning approaches.

**Fig. 4:**
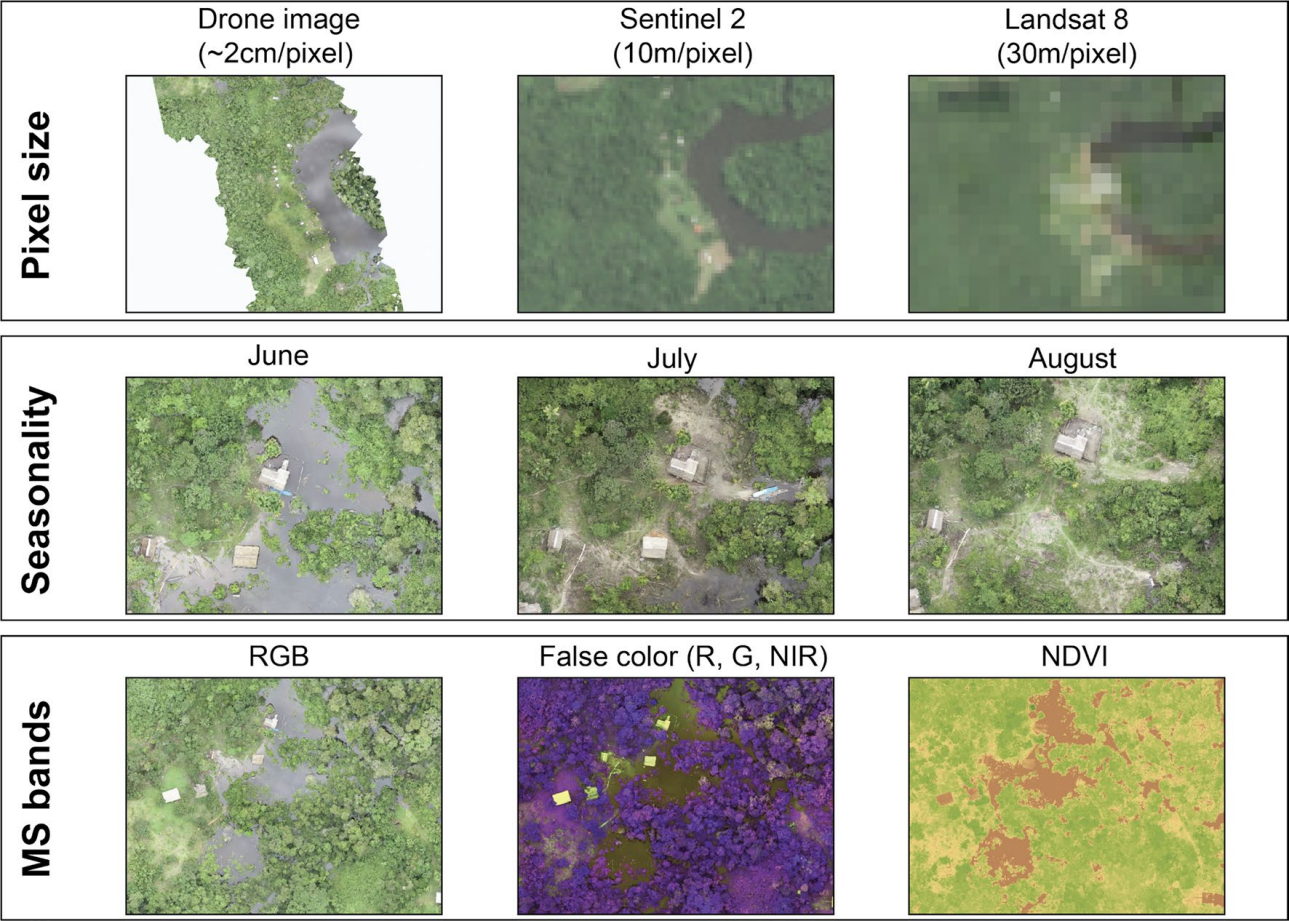
Drones are advantageous in their ability to detect small-sized features, as they produce high-resolution imagery at the sub-meter level. This is particularly important in vector-borne studies because water bodies/containers suitable for mosquito breeding are frequently small. The top row shows a comparison of the pixel size produced by a drone in contrast to two commonly used freely available satellite imageries. The middle row demonstrates changes in the landscape composition across a 2-month window, as captured by drones, which otherwise might be overlooked by satellite images. The bottom row shows imageries with common bands that are available in the sensor often used in drones: RBG cameras (left), multispectral cameras that have NIR and red edge band (middle) and an NDVI composite using red and NIR bands (right). MS, Mass spectrometry; NDVI, normalized difference vegetation index; NIR, near infrared; RBG, red, green and blue color model

The features to be identified and the analytical approach should determine selection of the drone sensor. Commercially available drone sensors include RGB, multispectral and thermal cameras and, increasingly, a range of active sensors, such as three-dimensional (3D) laser scanning (i.e. LiDAR) (Fig. 4). To collect data, the drone and sensor are flown over the AOI, typically in a grid-like configuration to allow collection of overlapping images. Using commercially available (e.g. Agisoft Metashape Professional; Agisoft LLC, St. Petersburg, Russia) or open-source (e.g. Open Drone Map) software, these images can then be processed to generate spatially referenced orthomosaics, which are images that have been stitched together and orthorectified. Additionally, photogrammetric methods (or Structure-from-Motion) can generate digital surface models and 3D representations of landscapes. These data can then be used as inputs into image analysis pathways or input directly into a Geographic Information System (GIS) software for viewing.

### Mapping juvenile aquatic habitats

Aquatic habitats used by malaria vectors have a spatially heterogeneous distribution that is controlled mostly by hydrological and geomorphological processes [54], together with regulated land use/land cover (LU/LC) [55, 56], resulting in a patchy distribution of water bodies, which are often spatially organized in clusters [57]. The distribution of aquatic habitats is also strongly correlated with rainfall, which can dictate the temporal dynamics of anopheline larval habitats and adult abundance [58–60]. Mapping the spatial distribution of aquatic habitats preferred by mosquitoes is crucial to the successful deployment of different control and surveillance practices. Also, targeting the most productive habitats could increase the cost-effectiveness of mosquito larval control programmes [61, 62].

**Fig. 5:**
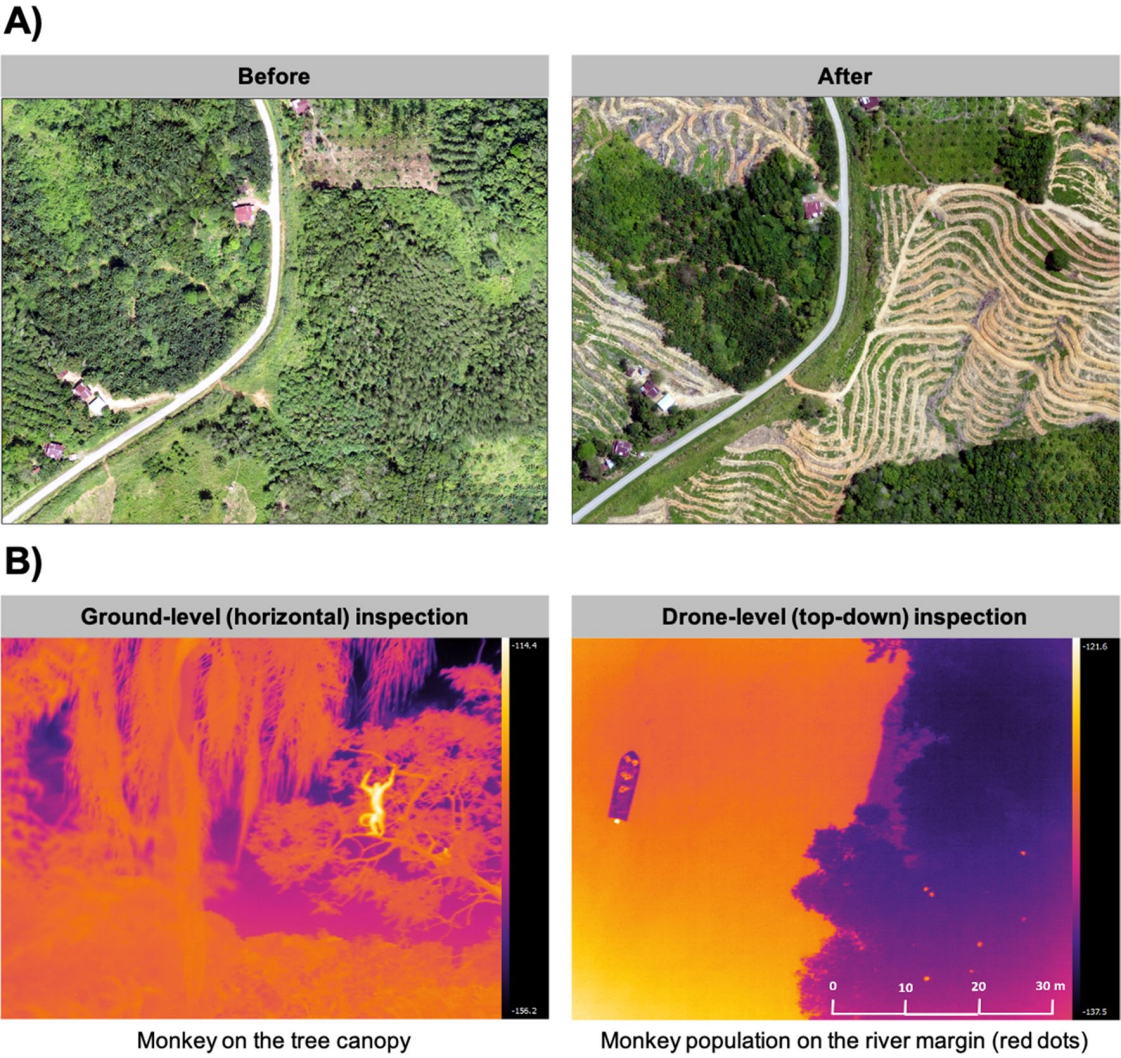
**a** Deforestation disrupts wildlife habitats and can bring human, mosquito and macaque populations in closer proximity, thereby increasing the potential for disease transmission. Monitoring wildlife populations is essential for understanding disease dynamics in these changing landscapes. **b** Inspection with thermal cameras

Remote sensing and geospatial mapping are realistic approaches to identify larval habitats that cover large areas, overcoming some operational challenges of conventional ground-based surveys [59, 63–65]. The identification of larval habitats using high spatial resolution satellite imagery may be challenging during the classification process, particularly when water bodies are small and turbidity is high [66, 67]. Another example of the use of remote sensing technology are malaria risk assessments through the study of anopheline larvae [68, 69]. Recently, object-oriented machine learning classification has been explored to overcome backscatter similarities of radar data when mapping open water or slightly vegetated areas between stretches of open water [70]. Proof-of-concept studies have proven the feasibility of using drones to map aquatic larval habitats of African [20, 71] and South American malaria vectors [22] (Table 1). The high-resolution imagery at relatively low cost compared with satellite images, the temporal flexibility to capture images by the drone user and the clear-sky conditions required for optical satellite imagery make this technology an effective operational tool for the identification of potential habitats [46] (Case study 2; Fig. 5).

**Table 1:** Examples of the use of uncrewed aerial vehicles in vector-borne diseases showing ecosystems, purpose of the study, mosquito species targeted and technical characteristics of the drone surveys

Location	Landscape/ecosystem	Area	Purpose	Feature of interest	Target vector	Equipment	Pixel resolution	Ground-based activities	Image processing	Analysis	Other	Outcomes	References
Westchester and Suffolk Counties, NY, USA	Suburban neighbourhoods	Westchester, 0.05 km^2^; Suffolk, 0.14 km^2^	Determine capabilities of UAVs to image preferred oviposition sites of *Aedes albopictus*; validate results by entomological survey	Oviposition containers	*Aedes albopictus*	DJI Mavic Pro drone (DJI, Shenzhen, China), with custom-built UAV designed around 3DR Pixhawk 1 flight controller (3DR, Berkeley, CA, USA) using a GoPro Hero 5 camera (GoPro, Inc., San Mateo, CA, USA)	DJI Mavic < 1 cm; Go Pro, 1.5—4 cm/pixel	Yes: entomological and container ground survey	Outer 500 pixels cropped to account for lens distortion with GO PRO photos using the open-source ImageMagick (2020); images sliced into 512 × 512-pixel images to translate to SSD network preinitialized weights and bounding box anchors; annotated aerial imagery (LabelImg) to determine container types UAVs could see; piexif in Python used to convert container locations into GPS	CNN based on the SSD300 algorithm using API Keras on top of Tensorflow. Amazon EC2 p2.xlarge instance handled computations using Amazon Machine Image	Flight at altitude of 50 m; pre-programmed flight paths (Ardu Pilot Development Team software suite 2019) for Pixhawk quadricopter and DJI GO 4 for Mavic Pro	CNN trained on UAV imagery detected up to 67% *Ae. albopictus* habitat; classified whole properties as positive or negative for larvae 80% of the time	[36]
Cote d'Ivoire	Rural, agricultural landscape	30.42 km^2^	To develop a technical workflow to integrate drone surveys and mosquito larval sampling. To characterize *Anopheles funestus* breeding sites in an agricultural setting	Large, semi-permanent water bodies	*Anopheles, Anopheles funestus*	DJI Phantom 4 Pro quadcopter drone, fitted with a DJI 4 K camera (8.8 mm/24 mm; f/2.8; 1″ CMOS sensor; 20 MP)	~ 4 cm	Yes: GPS coordinates and photos were collected via ODK, and the predominant land cover type was recorded	AgiSoft Metashape Pro (AgiSoft LLC, St. Petersburg, Russian Federation)	Images classified using collaborative online image labelling tool Groundwork (https://groundwork.azavea.com); developing of deep learning approaches (u-Net) integrating drone and satellite data on Amazon Web Services	Flight at an altitude of 150 m Flight plans were programmed using Pix4Dcapture and DJI GS Pro mapping applications	Development of land cover classification scheme with classes relative to *An. funestus* breeding ecology; developed protocols to integrate drone surveys and statistically rigorous entomological sampling	[113]
Maynas Province, Peru	Riverine/rainforest	~ 1 km^2^	Use of UAVs to identify breeding sites	Water bodies	*Nyssorhynchus darlingi *(formerly* Anopheles darlingi*)	DJI Phantom 4 Pro quadcopter drone, fitted with a DJI 4 K camera (8.8 mm/24 mm; f/2.8; 1'' CMOS; 20 MP) 3DR Solo (3DR) quadcopter fitted with a Parrot Sequoia multispectral sensor (Parrot SA, Paris, France)	DJI Phantom 4 Pro, GSD or spatial resolution of 0.1 m/pixel. 3DR Solo GSD of 0.02 m/pixel	31 water bodies located within 1 km of each of 4 villages surveyed were identified, characterized and sampled along edges for larvae using standard dippers	AgiSoft Photoscan Pro (AgiSoft LLC)	GEE. Integrated Development Environment at https://code.earthengine.google.com used to implement pixel-based random forest classification algorithm	DJI Phantom 4 Pro—altitude of ~ 100 m.3DR Solo drone was flown to an altitude of approximately 50 m	High-resolution multispectral imagery discriminated water bodies likely to be positive for* Ny. darlingi* with 86.73–96.98% accuracy (k-fold cross validation), with a moderate differentiation of spectral bands	[22]
Sabah, Malaysia/Palawan	Forest/agriculture	3 case study areas of 50–100 km^2^	To characterize land-use types and create a spatial sampling frame	Land cover	Habitats of *Anopheles balabacensis* and non-human primate reservoirs of *Plasmodium knowlesi*	senseFLY eBee drone (senseFLY, Cheseaux-sur-Lausanne, Switzerland), fitted with 16-megapixel digital camera, eMotion2 software (Ageagle Aerial Systems, Wichita, KS, USA)	Average resolution of 11.22 cm	Yes: ground-truth land cover classes; larval and adult mosquito surveys	Postflight Terra3D software (Terra3D, Paris, France)	Land cover classes manually digitized, used to identify training data for pixel-based classification of Landsat satellite data using random forest algorithms	350–400 m altitude	Use of UAVs most appropriate for detailed mapping of relatively small geographical areas where high-resolution satellite data are not readily available	[46]
Kinabatangan, Sabah, Malaysia	Riverine forest	10 × 1-km transects along 20 km of riverbank	To compare visual counting versus use of thermal cameras for primate census	8 diurnal primate species	N/A, reservoir	Custom built hexacopter drone with FLIR Thermal Camera (Teledyne FLIR LLC, Wisonville, OR, USA)		Yes: boat surveys of primates in early morning	FLIR Tools (Tledyne FLIR LLC Co.)	Visual identification and counting		Thermal cameras detected 1.78x (*P* < 0.001) more primates than were detected by eye; ground-truthing must be conducted during or immediately following to verify species, sexes, age classes and closely aggregated animals	[81]
Kasunga town, Malawi	Rural area surrounding artificial lakes in designated "humanitarian drone testing corridor"	8.9 km^2^ across 8 sites	Determining the feasibility of drone-led mosquito larval habitat identification in rural environments to inform local malaria control	Water bodies and aquatic vegetation	*Anopheles*	DJI Phantom 4 Pro quadcopter drone, fitted with NIR sensor(Sentera) vs. eBee SQ fixed-wing drone fitted with Parrot Sequoia multispectral camera (Parrot SA)	RGB camera: 3.3 for Phantom 4 Pro; 3.7 for eBee SQ; NIR sensor, 11 for both	Yes: larval surveys conducted concurrently with drone image capture; GPS co-ordinates recorded using ODK app; photographs taken using smartphone; aerial imagery were captured	Spervised classification of land cover classes (AgiSoft Metashape Pro; AgiSoft LLC)	Land cover and surface water layer classes were manually digitized, used to identify training data for pixel-based classification using random forest algorithms	flying at 120 m	Demonstration of potential for drone imagery as a tool to support identification of mosquito larval habitat in malaria endemic settings Cannot completely replace larval surveys	[71]

*CMOS* Complementary metal-oxide semiconductor,* CNN* convolutional neural network,* GEE* Google Earth Engine,* GPS* Global Positioning System,* GSD* ground sampling distance,* N/A* not available,* NIR* near infrared,* ODK* data collection tool,* RGB* red, green, blue colour model,* SSD* single-shot detector, *UAVs* uncrewed aerial vehicles

The proliferation of larval habitats of anopheline malaria vectors has been also associated with human activities, such as agriculture, settlement, mining or other landscape alterations that incorporate deforestation and vegetation clearance [72–75]. More frequent surveillance via drones will allow for the detection and subsequent development of quick responses to rapid changes and environmental alterations, such as urbanization, or changes in the use of rural/agricultural areas that might lead to new aquatic habitats favoured by certain mosquito species.

*Aedes*-borne disease surveillance programmes, such as those for dengue, chikungunya or Zika, commonly use periodic household inspections for detecting water-holding containers positive for *Aedes* larvae [76, 77]. Estimation of infestation indices, used to define and inform control actions, are labour-intensive in terms of vector intervention and public health staff and can be inaccurate due to variation in searching efforts, householder availability and/or mosquito egg-laying behaviour; they may also be intrusive for residents.

The centimetre-scale of the preferred oviposition habitats of *Ae. aegypti* and *Ae. albopictus* precludes the possibility of using high-resolution satellite imagery typically used for monitoring large water bodies for malaria vectors [20]. Approaches that leverage the high-pixel resolution of drone imagery have been explored to identify and map potential breeding sites characteristic of *Aedes*, such as cisterns, pots, tyres or flower pots. Ultimately, these approaches might support specific vector strategies, such as targeted application of larvicides against *Ae. aegypti* in, for example, rural areas of Central America [78] or guide preventative actions and prioritize programs that focus on controlling this species (e.g. in the city of Cuiabá, Brazil [79]).

In urban areas, drone imagery has also been used to locate preferred oviposition sites of *Ae. albopictus* [36]. In a study combining aerial imagery and entomological surveys in New York State (USA), up to 64% of the containers with water identified in ground surveys were detected with high-pixel resolution images and 14% were partially visible from the air (Table 1). In this suburban setting, detection of containers depended on the location within the property, with difficulty viewing those located on porches, trees, underneath awnings or near walls. Visibility was also affected by common features, such as tree cover and location of the drone when taking pictures [36].

#### Case study 1: Carrasco-Escobar et al. 2019 [22]

Carrasco-Escobar et al. [22] used drones mounted with regular RGB and multispectral cameras to collect high-resolution images to determine the spectral signature (characteristics of the light reflected from the land/water surface) of the most productive breeding sites of the malaria vector *Ny. darlingi* nearest to human habitation in Amazonian Peru. These researchers conducted a supervised classification approach (overall pixel-based accuracy: 86.73–96.98%) to identify water bodies and *Ny. darlingi*-positive and -negative areas within the water bodies identified, respectively, using machine learning techniques. They used a two-step process involving first the classification of several land type coverages and second the masking and classifying of only water bodies. The methodology and analysis were a proof-of-concept for testing whether improved larval source management can be combined successfully with LLINs and IRS to contribute to the elimination of transmission in malaria hotspots in the Amazon.

#### Case study 2: Stark et al. 2019 [80], Jumail et al. 2021 [81]

In addition to collecting data on mosquito habitats, drones are valuable for monitoring wider environmental factors influencing disease dynamics. Vector-borne diseases such as *Plasmodium knowlesi* and yellow fever are maintained by wild non-human primate reservoirs. Drones enable the mapping of fine-scale deforestation and other habitat modifications that influence movement patterns of wildlife populations [80]. Specifically, Jumail et al. [81] explored the use of thermal imagery to capture heat signatures of wild animals and estimate, in real-time, wild primate populations within changing landscapes in the Lower Kinabatangan Wildlife Sanctuary in Malaysian Borneo [81, 82]. These methods were validated initially by ground-based surveys prior to deploying aerial thermal surveys, with the objective of developing non-invasive techniques to assess wildlife populations and study disease dynamics.

## Vector control

As discussed in the previous section, drone application in mosquito vector control has been related mainly to larval source management (LSM). This strategy seeks to reduce the immature stages of malaria vectors using habitat modification or manipulation, larviciding or biological control [83]. When implemented well and incorporated into ongoing vector intervention programmes, LSM can reduce the densities of indoor and outdoor biting mosquitoes and help decrease the dependence on insecticides, and therefore help prevent the emergence of insecticide-resistant mosquitoes. However, WHO recommends larviciding only for special cases “when breeding sites are few, fixed and findable, and where the sites are easy to identify, map and treat” [62]. Such landscapes are uncommon not only in Africa but also in other malaria endemic regions like Southeast Asia and South America.

The use of drones in vector control can be divided into two main applications. The first is the use of the imagery to retrieve data that can serve as: (i) a source of visual inspection carried out before and after interventions; and (ii) as a communication tool, to detect water bodies and positive breeding sites and to create detailed maps of the mosquito breeding habitats and landscape. The second application is that drones can be used to disperse the intervention; for example, the dispersal of larvicide in liquid or granular form [84] and, more recently, as a potential means of deploying genetically modified mosquitoes [85]. For these activities, both the limited UAV flight time, which is largely dependent on battery capacity, and payload can be limiting factors [86, 87]. For instance, UAVs are especially effective in spraying when they are over water (e.g. rice paddies), on irregular or sloping ground, small areas and in places where it is hard to reach with other kinds of equipment. However, in units > 50 Ha, conventional ground and aerial sprayers proved to be more efficient [88]. Regarding flight time, the duration is even further shortened when the drones are fully loaded. Using a network of communicating drones, or swarms, could be one approach to overcome this operational challenge [89].

Some attempts have been made to use drones to deploy larvicides by leveraging the development of drones to apply pesticides for agriculture use [90]. For example, the dispersal of a biological control product to reduce juvenile malaria vectors in irrigated rice fields in East Africa [91] or as implemented in some mosquito control programmes for treating remote pools of standing water in brackish salt marsh with larvicides (Florida Keys Mosquito Control District). Using drones as deployment devices may overcome the challenges of treating and accessing large areas on foot and make aerial distribution affordable. Also, multispectral imagery collected with commercial drones has helped to predict the operational efficacy of some larvicide formulations against *Aedes vigilax* in complex environments with irregular canopy cover, such as mangroves [84].

In drone spraying operations, flight parameters might influence the effectiveness of droplet deposition when using liquid products and might need adjustments according to the larval habitat characteristics [92]. Flight height and velocity have a remarkable influence on the deposition amount [93]; high-speed rotation of the rotor—and of the air—rotor wind field [94]—and the downwash airflow may also affect intervention efficiency. Importantly, the use of drones in targeted areas could reduce harm to the environment by limiting wildlife disturbance in sensitive habitats [95, 96].

The use of drones in vector interventions represents a unique opportunity for the democratization of technology. One such organization, GLOBHE (https://globhde.com/about-us), uses crowddroning (sending local drone operators to capture ultra-high-resolution earth observation data) to determine appropriate interventions for vector-borne diseases and monitor local climate change, among other portfolios. A second example, WeRobotics, a not-for-profit (https://werobotics.org), empowers local experts in drone, data, artificial intelligence and robotics, creating knowledge hubs (of people) who then propose and activate local solutions for a wide range of health and environmental issues.

### Release of adult mosquitoes

Vector control approaches built on sterile insect techniques and *Wolbachia*-based strategies rely on mass-rearing and the dispersal of thousands of mosquitoes at a specific frequency depending on the nature of the strategy [97, 98]. Critical operational aspects need to be addressed before the deployment and scale-up of these interventions in the areas to be treated, such as aerial coverage, operational costs associated with the number of release sites and mosquito transportation [85, 99]. Operational programmes can benefit from using drones during the implementation of these interventions and to optimize some of the key technical aspects, including remote preparation of the material and then expanding release and dispersion areas.

The World Mosquito Program (WMP) has employed drones to optimize the release of *Wolbachia-*carrying mosquitoes for control of dengue virus on the island of Fiji, South Pacific. There, the development of an aerial release mechanism that can store up to 160,000 mosquitoes and release 200 every 50 m has proved to be much faster and more homogeneous than ground releases and also provided better coverage [100]. In another epidemiological scenario, in the fight against dengue and Zika in Brazil, Bouyer et al. [85] designed a mechanical compartment for dispersing sterile males by using drones. This study demonstrated minimum damage to mosquitoes and similar competitiveness between those released on the ground and by drone. Moreover, this drone-based dispersion modality was estimated to provide a 20-fold reduction in the estimated costs of implementing the sterile insect technique (SIT).

## Challenges and technological developments

The drone industry is expanding and gaining increasing interest from a wide range of stakeholders for use in civil applications [101]. As discussed above, important developments in the industry have been focussing on safety improvements. In this section, we detail additional challenges to drone operation, as well as recent technological developments for control and surveillance of vector-borne diseases.

### Capacity building

Most countries require operator training for authorization of drone operations. However, there is still no consensus on the minimum levels of training ensuring safe flight and ground operations [102]. Despite algorithms that have been developed recently to automate common human tasks related to drone operations, overtrust and automation bias in autonomous systems could be extremely dangerous in safety–critical settings [103]. Recently, Kucherov et al. [104] proposed and evaluated a training process for specialists in the maintenance and operation of UAVs; however, this field is still under development.

### Availability of training data

Accuracy of supervised image classification to detect breeding sites is largely dependent on the availability of training data, i.e. identified data of the features of interest used to fit predictive models [105, 106]. Recent initiatives have focussed on developing large training datasets of such features, including roads, housing or agricultural land types [107, 108]. These datasets are typically labelled using standardized methodologies, such as the Spatiotemporal Asset Catalog [109], enabling utilization by different platforms for various purposes. However, there is no standard image repository to share mosquito habitat training data, and most projects need to collect their own.

### Energy management

One important challenge of drone missions is the current trade-off between battery weight and flight duration, as increased weight limits the flight duration. To cover large areas, multiple returns to the charging station are needed, a potential problem for drone operations in rural areas. Current developments on wireless charging [110, 111] and lightweight solar-powered battery components [112] may improve drone operations*.*

### Evaluation of performance

There is a critical need to evaluate the impact of drones on the control or surveillance of vector-borne diseases using metrics appropriate for the intended end use. While model validation is an important step of image analysis, new approaches are needed to assess impacts on disease transmission and efficacy of control programmes. This may involve identifying target levels of sensitivity and specificity for detecting or treating specific features, similar to practices used to evaluate diagnostic methods.

## Conclusions

In areas with endemic and residual transmission of vector-borne diseases, effective vector control measures must be considered for moving towards elimination or, at the very least, dramatic reduction of pathogen transmission. A major consideration in this context, for malaria control, is targeting outdoor transmission through the identification, surveillance and treatment of aquatic habitats favourable for juvenile vector mosquito stages. Recent developments in drones (or UAVs) can be effective for accurately conducting surveillance, assessing habitat suitability for larval and/or adult mosquitoes and implementing interventions. Examples that leverage this technology include high-resolution mapping of water bodies in the endemic areas in Latin America, Southeast Asia and Africa. The implementation of this technology for current control activities requires specific considerations in terms of local regulations, safety, privacy and community acceptance. In this rapidly evolving field, emerging technological developments are addressing these concerns. However, despite the rapidly increasing availability of this technology, drones remain best used in conjunction with other existing approaches, such as field surveys, analysis of satellite-based EO data and deployment of control measures. Drones have the potential to improve and target these existing activities, adding to a growing suite of tools for vector control.

## Supplementary Information

**Additional file 1: Text 1. **Glossary.

## Data Availability

Not applicable.

## Abbreviations

AOI: Area of interest
EO: Earth observation
IRS: Indoor residual spraying
LiDAR: Light detection and ranging
LLINs: Long-lasting insecticidal nets
LSM: Larval source management
LU/LC: land use/land cover
RADAR: Radio detection and ranging
RGB: Red, green, blue colour model
SIT: Sterile insect technique
UAVs: Uncrewed aerial vehicle
WMP: World mosquito programme
