# Supplementary material for: The use of drones for mosquito surveillance and control
Source: Parasit Vectors. 2022 Dec 16;15:473. doi: 10.1186/s13071-022-05580-5 (PMC9758801; doi:10.1186/s13071-022-05580-5)
Supplement: Supplementary file 1 — Additional file 1: Text 1. Glossary. [file 13071_2022_5580_MOESM1_ESM.docx]

**The use of drones for mosquito surveillance and control**

Gabriel Carrasco-Escobar^1,2^*, Marta Moreno^3^, Kimberly Fornace^3, 4^, Manuela

Herrera-Varela^5^, Edgar Manrique^1^, and Jan E. Conn^6,7^

**Author Affiliated Institutions:**

^1^ Health Innovation Laboratory, Institute of Tropical Medicine “Alexander von Humboldt”, Universidad Peruana Cayetano Heredia, Lima, Peru

^2^ School of Public Health, University of California San Diego, USA

^3^ Faculty of Infectious and Tropical Diseases and Centre for Climate Change and Planetary Health, London School Hygiene and Tropical Medicine, London, UK

^4^ Institute of Biodiversity, Animal Health and Comparative Medicine, University of Glasgow, Glasgow, UK

^5^ Grupo de Investigación en Entomología, Facultad de Medicina, Universidad Nacional de Colombia, Bogotá D.C., Colombia

^6^ The Wadsworth Center, New York State Department of Health, Albany, New York, USA

^7^ Department of Biomedical Sciences, School of Public Health, State University of New York at Albany, NY, USA.

*** Corresponding Author:** [gabriel.carrasco@upch.pe](mailto:gabriel.carrasco@upch.pe)

Email contacts:

Gabriel Carrasco-Escobar: [gabriel.carrasco@upch.pe](mailto:gabriel.carrasco@upch.pe)

Marta Moreno: [marta.moreno@lshtm.ac.uk](mailto:marta.moreno@lshtm.ac.uk)

Kimberly Fornace: [kimberly.fornace@lshtm.ac.uk](mailto:kimberly.fornace@lshtm.ac.uk)

Manuela Herrera-Varela: [manuelahv82@gmail.com](mailto:manuelahv82@gmail.com)

Edgar Manrique: [edgar.manrique@upch.pe](mailto:edgar.manrique@upch.pe)

Jan E.Conn: [jan.conn@health.ny.gov](mailto:jan.conn@health.ny.gov)

**Keywords:** Drones, Unmanned Aerial Vehicle, Malaria, Dengue, control, infectious diseases

# **Additional file 1**

# **Glossary**

**Area of Interest (AOI) / Region of Interest (ROI):** The geographic extent used to define a focus area for either a map or database production. The area/region of interest could be based on an item in an index or a polygon feature.

**Drone:** (also known as Unmanned Aerial Vehicle or uncrewed aerial vehicle – UAV). This is an aircraft without a human pilot on board that can fly autonomously or be controlled remotely. The two main drone types are *fixed-wing* and *multirotor drones*.

**Earth Observation (EO):** Earth observation is the gathering of information about planet Earth’s physical, chemical and biological systems via remote sensing technologies, usually involving satellites carrying imaging devices [50]. Examples include: EOSDIS from NASA, European Space Agency, European Earth Observation Programme (Copernicus), and the Global Earth Observation System of Systems (GEOSS).

**Ground truthing:** The accuracy of remotely sensed or mathematically calculated data based on data measured in the field. In remote sensing, it is especially important to relate image data to real features and materials on the ground. The collection of ground truthed data enables calibration of remote-sensing data, and aids in the interpretation and analysis of what is being sensed.

Ground truthing is especially important in the initial supervised classification of an image. When the identity and location of land cover types are known through a combination of field work, maps, and personal experience these areas are known as training sites. The spectral characteristics of these areas are used to train the remote sensing software using decision rules for classifying the rest of the image.

**Image classification:** The process of classifying image pixel data from raw remote sensing into different categories, labels or land cover types.

**Indoor Residual Spraying (IRS):** A malaria vector control intervention that involves coating the walls and other surfaces of a house with a residual insecticide [114].

**Larval Source Management (LSM):** This is the management of aquatic habitats (water bodies) that are potential larval habitats for mosquitoes, in order to prevent the completion of development of the immature stages. LSM is only recommended as a supplementary malaria vector control measure and comprises temporal or permanent habitat modification and manipulation, larviciding and/or biological control [61, 62].

**Long Lasting Insecticidal Nets (LLINs):** Nets treated with an insecticide incorporated into the net fabric, which retains the insecticide activity up to three years, even after repeated washing. Mass distribution of LLINs has been the primary vector control tool for malaria-endemic countries for the last 20 years [4].

**Machine (Artificial) Intelligence (AI)/ Deep Learning:** Machine learning is a branch of artificial intelligence focused on the study of algorithms which learn from data and automatically improve over time. Algorithms can be used to identify patterns from imagery and are widely used to classify EO data. Deep learning is a type of algorithm which uses artificial neural networks and mimics the brain to process data and identify complex features.

**Multispectral image Camera (MSC):** A multispectral image camera captures image data within specific wavelength ranges in narrow bands across the electromagnetic spectrum.

**Normalized Difference Vegetation Index (NDVI):** NDVI quantifies vegetation and is an indicator of photosynthetically active biomass. It is calculated as a ratio between the absorbed red light (R) and the reflected near infrared (NIR) values (NIR-R)/(NIR+R).

**Orthomosaic**: A georeferenced mosaic of overlapped images which includes correction for topographic distortions for each AOI or community.

**Orthorectification**: Adjustment for the topographical features of the Earth’s surface and tilts of aerial or satellite sensors.

**Parcels**: Basic spatial units used for classification.

**Spatial resolution:** This is defined by the size of each pixel within a digital image and the area on the Earth’s surface represented by that pixel, i.e., represents the area on the ground that each pixel covers. The finer the resolution, the more detail that can be seen.

**Spectral resolution:** This is the ability of a sensor to discriminate finer wavelengths, i.e., having more and narrower bands. Sensors with 3 to 10 bands are considered to be multispectral, and with hundreds or even thousands of bands, hyperspectral. The narrower the range of wavelengths for a given band, the finer the spectral resolution.

**Radiometric resolution:** This is the amount of information in each pixel or the number of bits representing the energy recorded and each bit records an exponent of power 2. The higher the radiometric resolution, the more values are available to store information, and greater detail and variation in light can be distinguished.

**Raster data:** Data stored in a grid of cells (or pixels) that contain information that represent a real-world phenomenon (i.e., climate or land cover).

**Remote sensing:** Process of scanning and detecting physical characteristics of an area from a distance. Earth Observation refers to remote sensing specifically used to characterise the Earth.

**Remote sensors:** Remote sensing instruments (sensors) are of two primary types according to the source of signal they use to explore the object. *Active sensors* operate with their own source of energy to illuminate the objects they observe. *Passive sensors* detect natural energy (radiation) reflected from the target. Reflected sunlight is the most common source of radiation measured by passive sensors.

*Passive sensors* include different types of radiometers and spectrometers, and most of them operate in the visible, infrared, thermal infrared, and microwave portions of the electromagnetic spectrum. Accelerometer, hyperspectral radiometer and spectrometer are some examples of passive remote sensors.

The majority of the *active sensors* operate in the microwave portion of the electromagnetic spectrum, allowing for penetration of the atmosphere under most conditions. As they do not require sunlight, active sensors function at any time of the day and they are relatively independent of atmospheric scatterings. Radar, a sensor used for detecting and tracking radio signals, and LiDAR, a light detection and ranging sensor which determines distance to the object by using the speed of light to calculate the distance travelled, are some examples of active sensors [50].

**RGB image:** Also known as true colour image, these are regular images that use an additive colour model based on red (R), green (G), and blue (B).

**Temporal resolution:** The temporal resolution corresponds to the frequency of revisits over a site and is dependent on the orbitography of the platform or satellite on which the sensor is hosted, the sensor characteristics and the swath width.

**Thermal imaging:** Also called infrared thermography, this is imaging based on thermal radiation. The thermal radiation emitted by most objects is in the mid- to far-infrared spectral region.

**Vectorial capacity:** This is defined as the average number of inoculations with a specified parasite, originating from one case of malaria per unit time that the population would distribute if all the female vectors that take a bloodmeal from the initial case become infected [115].

**Unmanned Aerial/aircraft System (UAS):** This includes a UAV, a ground-based controller and a system of communication between the two elements.
